# 6-Phosphogluconolactonase Promotes Hepatocellular Carcinogenesis by Activating Pentose Phosphate Pathway

**DOI:** 10.3389/fcell.2021.753196

**Published:** 2021-10-26

**Authors:** Changzheng Li, Jie Chen, Yishan Li, Binghuo Wu, Zhitao Ye, Xiaobin Tian, Yan Wei, Zechen Hao, Yuan Pan, Hongli Zhou, Keyue Yang, Zhiqiang Fu, Jingbo Xu, Yanan Lu

**Affiliations:** ^1^Department of Anesthesiology, Sun Yet-sen Memorial Hospital, Sun Yet-sen University, Guangzhou, China; ^2^Key Laboratory of Stem Cells and Tissue Engineering, Ministry of Education, Zhongshan School of Medicine, Sun Yat-sen University, Guangzhou, China; ^3^Department of Pancreaticobiliary Surgery, Sun Yat-sen Memorial Hospital, Sun Yat-sen University, Guangzhou, China; ^4^Key Laboratory of Malignant Tumor Gene Regulation and Target Therapy of Guangdong Higher Education Institutes, Sun Yat-sen Memorial Hospital, Sun Yat-sen University, Guangzhou, China; ^5^Department of Hematology, The Fifth Affiliated Hospital, Sun Yat-sen University, Zhuhai, China

**Keywords:** hepatocellular carcinoma, 6-phosphogluconolactonase, metabolic reprogramming, pentose phosphate pathway, ROS

## Abstract

Hepatocellular carcinoma (HCC) has a poor prognosis due to the rapid disease progression and early metastasis. The metabolism program determines the proliferation and metastasis of HCC; however, the metabolic approach to treat HCC remains uncovered. Here, by analyzing the liver cell single-cell sequencing data from HCC patients and healthy individuals, we found that 6-phosphogluconolactonase (PGLS), a cytosolic enzyme in the oxidative phase of the pentose phosphate pathway (PPP), expressing cells are associated with undifferentiated HCC subtypes. The Cancer Genome Atlas database showed that high PGLS expression was correlated with the poor prognosis in HCC patients. Knockdown or pharmaceutical inhibition of PGLS impaired the proliferation, migration, and invasion capacities of HCC cell lines, Hep3b and Huh7. Mechanistically, PGLS inhibition repressed the PPP, resulting in increased reactive oxygen species level that decreased proliferation and metastasis and increased apoptosis in HCC cells. Overall, our study showed that PGLS is a potential therapeutic target for HCC treatment through impacting the metabolic program in HCC cells.

## Introduction

Hepatocellular carcinoma (HCC) is the third leading cause of cancer-related death worldwide (Jiang et al., 2000; Imamura et al., 2003; Ye et al., 2016; Yang et al., 2019). HCC usually occurs in patients with chronic liver diseases related to viral infection (chronic hepatitis B and C viruses), alcoholism (alcohol and aflatoxin), and liver metabolic disorders (diabetes and non-alcoholic steatohepatitis) (Ganne-Carrie and Nahon, 2019; Kanwal and Singal, 2019). Hepatectomy and liver transplantation are the two main treatments for HCC currently, but the HCC recurrence rate is high because of the easy metastasis of liver cancer cells (Clavien et al., 2012; Sapisochin and Bruix, 2017; Yoshida et al., 2019). Hence, limited cognition hinders clinical treatment of HCC. In order to seek effective clinical treatment for HCC, more knowledge about HCC is necessary.

HCC requires metabolic reprogramming for continuous growth and rapid proliferation (Cancer Genome Atlas Research Network, 2017; Tian et al., 2019). Hepatocytes mainly produce ATP through oxidative phosphorylation (OXPHOS), whereas HCC cells produce ATP through anaerobic glycolysis, instead of OXPHOS (Feng et al., 2020). The pentose phosphate pathway (PPP) is a metabolic pathway parallel to glycolysis (Jiang et al., 2014; Patra and Hay, 2014; Wu et al., 2018). The PPP pathway consumes intermediate glucose 6-phosphate (G6P) through the oxidized and non-oxidized branches to produce fructose 6-phosphate and glyceraldehyde 3-phosphate (Ma et al., 2020). The PPP pathway metabolites, ribose 5-phosphate (R5P) and NADPH, are mainly produced by rate-limiting enzymes glucose-6-phosphate dehydrogenase (G6PD) and 6-phosphogluconolactonase (PGLS), which are essential for the survival of HCC cells and the synthesis of fatty acids (Gao et al., 2019; Jing et al., 2019; Li M. et al., 2019; Ghergurovich et al., 2020). Emerging evidence has demonstrated that G6PD is involved in the occurrence of HCC, but the role of PGLS in HCC remains unclear. PGLS, a hydrolase, specifically catalyzes the hydrolysis of 6-phosphogluconolactone to 6-phosphogluconic acid (Beutler et al., 1985).

In our study, by analyzing the liver single-cell RNA sequencing data from HCC patients or healthy individuals, we found that PGLS was highly expressed in undifferentiated HCC cells, and down-regulation of PGLS *in vitro* could inhibit the proliferation, migration, and invasion of HCC cells. In addition, PGLS has a new tumor-promoting effect in HCC by activating the PPP pathway.

## Materials and Methods

### Patients and Tissue Samples

A total of six pathologically diagnosed HCC tissues and matched tumor-adjacent tissues were obtained from patients at the Third Affiliated Hospital of Sun Yat-sen University. The use of clinical samples was approved by the ethics committee of the Third Affiliated Hospital of Sun Yat-sen University, and written informed consents were obtained from all enrolled patients. All patients did not receive preoperative therapies.

### Cell Culture and Transfection

HCC cell lines (Hep3b, Huh7) were purchased from the Cell Bank of Type Culture Collection of Chinese Academy of Sciences (Shanghai, China). All cells were maintained in a six-well plate in RPMI-1640 medium (Corning, 10-040) supplemented with 10% fetal bovine serum (FBS) (Hyclone, SH30084), 100 μg/mL streptomycin/penicillin (Hyclone, SV30010) in a humidified 37°C incubator with 5% CO_2_. The PGLS siRNA (siPGLS) was designed and obtained from Gene Pharma (Guangzhou, China). The transfection assay was carried out using Lipofectamine 2000 (Invitrogen, 11668030) following the protocols. The culture media was supplemented with 6-aminonicotinamide (6-ANA) (10 μM, Target-mol, T7545) 48 h.

### Quantitative Real-Time Polymerase Chain Reaction

Total RNA was extracted from HCC cell lines or clinical samples using a TRIZOL reagent (Magen, R4801-02) according to the manufacturer’s instructions. Quantitative real-time polymerase chain reaction (PCR) was performed using SYBR Green PCR kit protocol (Bio-Rad, 1725150). PCR primer sequences are listed in Supplementary Table 1.

### Western Blot Analysis

The same number of cells from each population to be analyzed was sorted into phosphate-buffered saline (PBS) with 2% FBS. The cells were washed with PBS and lysed by RIPA. Equal amounts of protein extracts were fractionated by 12.5% sodium dodecyl sulfate–polyacrylamide gel electrophoresis and transferred to a polyvinylidene fluoride membrane (IPVH00010, Merck Millipore). After blocking with 5% non-fat milk in Tris-buffered saline with Tween-20 (TBST, pH 7.6) for 1 h at room temperature, the membranes were incubated with primary antibodies including anti-PGLS (rabbit, 1:1,000, GTX120327, Genetex), anti-CK18 (rabbit, 1:1,000, 10830-1-AP, Proteintech), and anti–β-actin (rabbit, 1:1,000, 4970s, Cell Signaling Technology) overnight at 4°C and then incubated with secondary antibodies (rabbit, 1:10,000, W401B, Promega) for 1 h at room temperature, which was detected by digital imaging with a charge-coupled device camera system (Odyssey Fc). The images shown are representative of images from at least three experiments.

### Flow Cytometry

For apoptosis, the cells were fixed, permeabilized, and stained by Tunel Detection kit (C1086, Beyotime) according to manufacturer’s instructions. For reactive oxygen species (ROS) activity analysis, the cells were stained by 5 μM DCFDA (D6883, Sigma). Cell sorting and analysis were performed using an Attune NxT analyzer (Thermo Fisher Scientific) or InFlux Cell Sorter (BD Biosciences). Data analysis was performed using FlowJo software.

### Metabolic State Analysis

PGLS^high^ and PGLS^low^ cells were sorted and then lysed; intracellular NADP^+^/NADPH ratio was measured using the NADP^+^/NADPH Assay Kit (KA1663, Abnova) according to the manufacturer’s instructions.

### Cell Proliferation Assay

HCC *in vitro* proliferation was measured by calcein-AM/PI kit (C2015S, Beyotime) according to the manufacturer’s instructions.

### Transwell Assay

Hep3b and Huh7 cells were added into the upper chambers of Matrigel-uncoated (cell migration) or coated (cell invasion) Transwells (ET BIOFIL, Guangzhou, TCS004024). The lower chambers were added medium with 10% FBS, and the upper chambers were serum-free medium. After 24 h culture, the migrated or invaded cells (on the bottom of the filters) were fixed using 4% paraformaldehyde (Mei Lun, China, MA0192) and stained with 0.5% crystal violet for 1 h. The number of migrated or invaded HCCs was counted under a light microscope by randomly selecting five fields.

### Colony Formation Assay

A total of 5 × 10^4^ Hep3b and Huh7 cells were plated into six-well plates. Colonies were fixed with 4% paraformaldehyde and stained with crystal violet (Beyotime, Shanghai, C0121-100ML for 30 min at room temperature. The visible colonies were counted manually.

### Wound-Healing Assay

A total of 5 × 10^4^ Hep3b and Huh7 cells were seeded into six-well plates and grown to 80% cell abundance. Then, a single layer wound was created using a pipette tip, and we took images (Olympus, BX51). Imaging was repeated at the same location and further analyzed by ImageJ software. All assays were conducted three times.

### scRNA-Seq Data Processing

Raw genomic data have been deposited in the Gene Expression Omnibus database with accession number GSE149614. The scRNA-seq data are available from the corresponding author upon reasonable request; 28,687 non-tumor liver cells and 34,414 primary tumor cells from 10 HCC patients were included. Normalization, dimensionality reduction, and clustering were performed with the Seurat 3.2.3 R package (Butler et al., 2018) on RStudio. Cells were filtered to have > 500 and < 5,000 detected genes and < 5% of total UMIs mapping to the mitochondrial genome. Data set normalization was performed by dividing the UMI counts per genes by the total UMI counts in the corresponding cells and log-transforming, and following the results, scaling and centering. Cells underwent dimensionality reduction with the uniform manifold approximation and projection method (UMAP). HCC-like clusters were selected by HCC markers including GPC3, CD24, and MDK (Tsuchiya et al., 2015; Lu et al., 2018; Yu et al., 2018). Feature plots were generated by the Seurat function feature plot. Pseudotime trajectory was analyzed by monocle2 on basis of the Seurat clustering (Subramanian et al., 2005; Qiu et al., 2017). Signature genes of each cluster were obtained using the Seurat function FindMarkers with “wilcox” test. Venn plots were generated by Venn Diagram R packages. Kyoto Encyclopedia of Genes and Genomes (KEGG) analysis and plots were performed using cluster Profiler and ggplot2 R package. Gene lists were preranked by the fold change values of the differential expression analysis using Seurat. Gene sets were obtained from Gene Ontology database as indicated. Heatmap was generated by the pheatmap R package.

### Statistical Analyses

Data are expressed as means ± standard deviation (SD). All experiments were analyzed by Student *t*-test, and differences were considered statistically significant if *p* < 0.05. Differences were considered statistically significant if *p* < 0.05, ^∗^*p* < 0.05, ^∗∗^*p* < 0.01, ^∗∗∗^*p* < 0.001.

## Results

### 6-Phosphogluconolactonase Was Specifically Highly Expressed in Human Hepatocellular Carcinoma Samples

**FIGURE 1:**
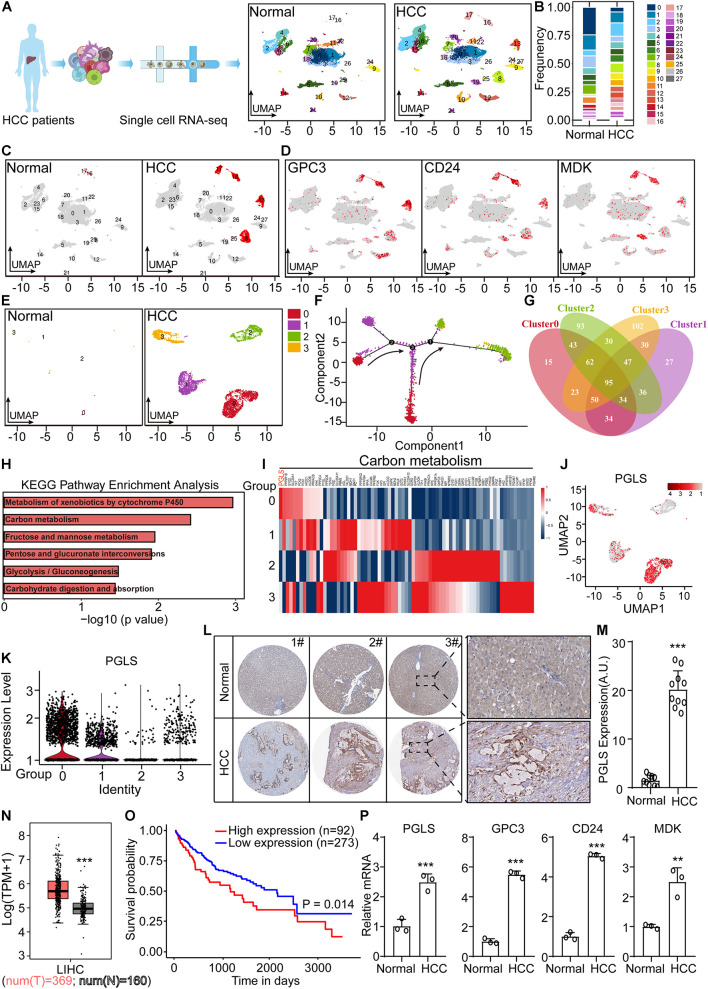
PGLS was specifically highly expressed in human HCC samples. **(A)** The UMAP dimensionality reduction results of scRNA-seq data from non-tumor and HCC patients. **(B)** The frequency of cells in each cluster. **(C)** Four more clusters presented in HCC samples. **(D)** These four cell clusters were labeled by HCC markers (GPC3, CD24, and MDK). **(E)** Four clusters specifically presented in HCC samples. **(F)** The pseudotime trajectory analysis was done for the above four clusters, which showed that cluster 0 was the most primitive. **(G)** The overlapped marker genes among the new four clusters. **(H)** KEGG enrichment using the specifically high expression genes in new cluster 0. **(I)** High expression genes in new cluster 0, in carbon metabolism. **(J)** The expression of PGLS in the UMAP plot. **(K)** The expression of PGLS in the violin plot. **(L)** The expressions of PGLS in normal and HCC samples were detected by IHC staining. **(M)** The quantitative results of IHC staining in normal and HCC samples. **(N)** PGLS mRNA expressions between HCC tissue (*n* = 369) and non-tumor liver tissue (*n* = 160) of TCGA and GTE database. **(O)** The survival curve for the HCC patients. **(P)** The differential expressed marker genes between non-tumor and HCC samples. Data show individual values and mean ± SD. m, n, and *p*, unpaired two-tailed Student *t*-tests, assessed statistical significance, **p < 0.01, ***p < 0.001.

From the Gene Expression Omnibus database, we downloaded scRNA-seq data of non-tumor and HCC patient liver cells. In total, 28 clusters were shown after UMAP dimensionality reduction (Figures 1A,B). We found that clusters 8, 13, 16, and 17 were specifically presented in patient samples, labeled by HCC markers (GPC3, CD24, and MDK) (Figures 1C,D). Then we extracted these four clusters for pseudotime trajectory analysis, which showed that cluster 0 was the most primitive (Figures 1E,F). Venn plot presented the overlapped marker genes among the new four clusters (Figure 1G). The specifically high expression genes, in cluster 0, were used for KEGG enrichment analysis. It showed that several top pathways were associated with carbon metabolism (Figure 1H). Next, we found the expression of PGLS in carbon metabolism was the highest (Figure 1I). Consistent with this, the UMAP plot and violin plot showed specific high expression level of PGLS in new cluster 0 (Figures 1J,K). Similar rising level could be observed in human HCC samples from the results of IHC staining and The Cancer Genome Atlas (TCGA) (Figures 1L–N). Compared with the survival probability of the high PGLS expression group, the low PGLS expression group showed a longer survival period (Figure 1O). PGLS transcripts were profoundly higher in human HCC samples by quantitative PCR. Similar rising levels could be observed in the HCC markers GPC3, CD24, and MDK (Figure 1P).

### 6-Phosphogluconolactonase Pathway Led to Significant Activation of Pentose Phosphate Pathway in Hepatocellular Carcinoma

**FIGURE 2:**
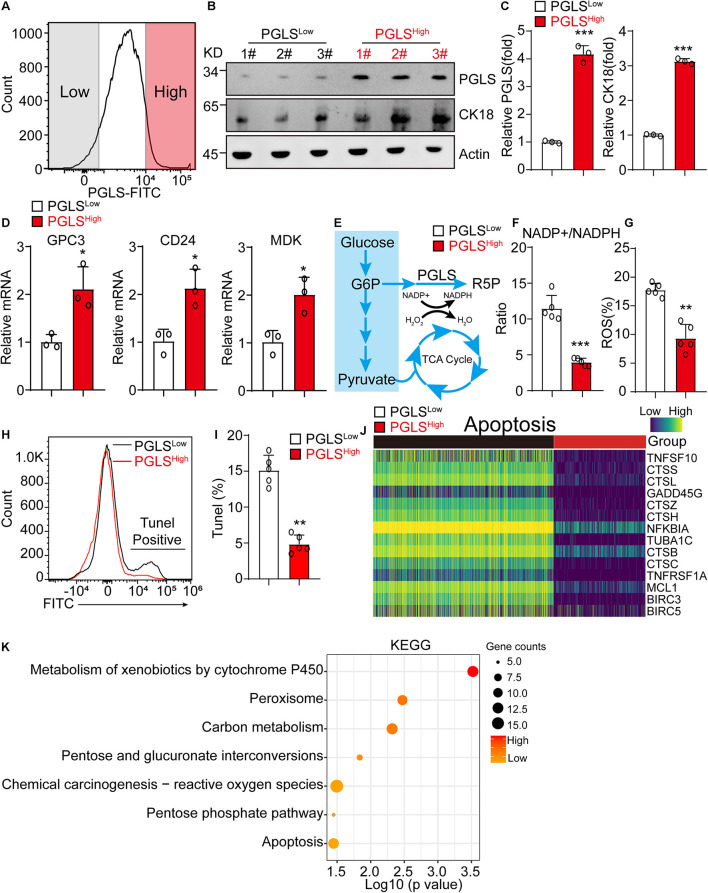
PGLS pathway led to significant activation of PPP in HCC. **(A)** The representative FACS plot of PGLS^high^ and PGLS^low^ HCC cells. **(B,C)** Western blot of the PGLS and CK18 in PGLS^high^ and PGLS^low^ cells from human HCC patients. β-Actin is a loading control. **(D)** The relative expression of HCC marker genes in PGLS^high^ and PGLS^low^ cells (*n* = 3 replicates). **(E)** Glycometabolism diagram. **(F)** The NADP^+^/NADPH ratio in PGLS^high^ and PGLS^low^ cells (*n* = 5 replicates). **(G)** The percentage of ROS in PGLS^high^ and PGLS^low^ cells (*n* = 5 replicates). **(H)** The representative FACS plot of PGLS^high^ and PGLS^low^ cells for apoptosis analysis. **(I)** The apoptosis rate of PGLS^high^ and PGLS^low^ cells. **(J)** The expression profile of genes associated with apoptosis pathway between PGLS^high^ and PGLS^low^ cells. **(K)** KEGG analysis of PGLS^high^ and PGLS^low^ cells. Data show individual values and mean ± SD. **(C,D,F,G,I)** Unpaired two-tailed Student *t*-tests, assessed statistical significance, *p < 0.05, **p < 0.01, ***p < 0.001.

HCC cells from human liver cancer tissues were sorted by the expression level of PGLS for further exploring the difference between these two groups. PGLS was also highly expressed in PGLS^high^ cells (Figure 2A), with a higher expression level of CK18, which was used for HCC diagnosis in clinic (Figures 2B,C). PGLS^high^ cells presented a higher transcription level of GPC3, CD24, and MDK (Figure 2D). It also showed a lower NADP^+^/NADPH ratio, which was associated with the reduction of ROS production and apoptosis (Figures 2E–I), we next used scRNA-seq data to investigate the role of PGLS in HCC; we found that the expression of apoptosis-related genes was negative correlated with the expression of PGLS (Figure 2J). Furthermore, this kind of apoptosis occurs because of the activation of ROS relative signaling pathways (metabolism of xenobiotics by cytochrome P450, chemical carcinogenesis–ROS) (Figure 2K).

### 6-Phosphogluconolactonase Regulated the Proliferation and Apoptosis of Hepatocellular Carcinoma

**FIGURE 3:**
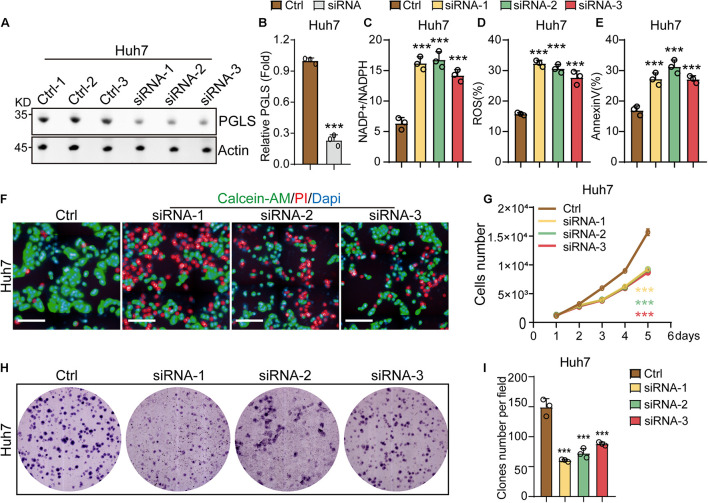
PGLS regulated the proliferation and apoptosis of HCC. **(A)** Transfection with the three siRNAs could effectively knock down the expression of PGLS on Huh7. **(B)** Quantitative analysis of Western blot in **(A)**. **(C)** The NADP^+^/NADPH ratio on Huh7. **(D)** The percentage of ROS on Huh7. **(E)** The apoptosis rate on Huh7. **(F)** Fluorescence micrographs of Huh7 after staining with calcein-AM, PI, and DAPI. **(G)** Proliferation curve of Huh7 transfected by siRNA-1, siRNA-2, and siRNA-3. **(H)** Representative field of CFUs, formed by Huh7 treated with siRNA-1, siRNA-2, and siRNA-3. **(I)** CFU number per field on Huh7. Data show individual values and mean ± SD. **(B–E,G,I)** Unpaired two-tailed Student *t*-tests, assessed statistical significance, ***p < 0.001.

To investigate the effects of the rate-limiting enzymes of PGLS in HCC cell lines, Huh7 and Hep3b cells were transfected with siRNA-1, siRNA-2, or siRNA-3. We found that all three siRNAs could reduce the expression level of PGLS in Huh7 cell lines as compared to control groups (Figures 3A,B). Similar results could be observed in Hep3b cell lines (Supplementary Figures 1A,B). In addition, we found that the treatment of siRNA-1, siRNA-2, and siRNA-3 increased the NADP^+^/NADPH ratio in HCC cell lines (Figure 3C and Supplementary Figure 1C). Meanwhile, ROS and apoptosis rate in the three knockdown (KD) groups were also significantly higher as compared to the control group (Figures 3D,E and Supplementary Figures 1D,E). After transfection by siRNA-1, siRNA-2, and siRNA-3, both HCC cell lines presented a slower proliferation rate at all six time points (Figures 3F,G and Supplementary Figures 1F,G). Consistent with this, Huh7 and Hep3b cells showed a significantly smaller size and fewer numbers of CFUs (colony-forming units) as compared to the control group (Figures 3H,I and Supplementary Figures 1H,I).

### 6-Phosphogluconolactonase Regulated the Migration and Invasion of Hepatocellular Carcinoma

**FIGURE 4:**
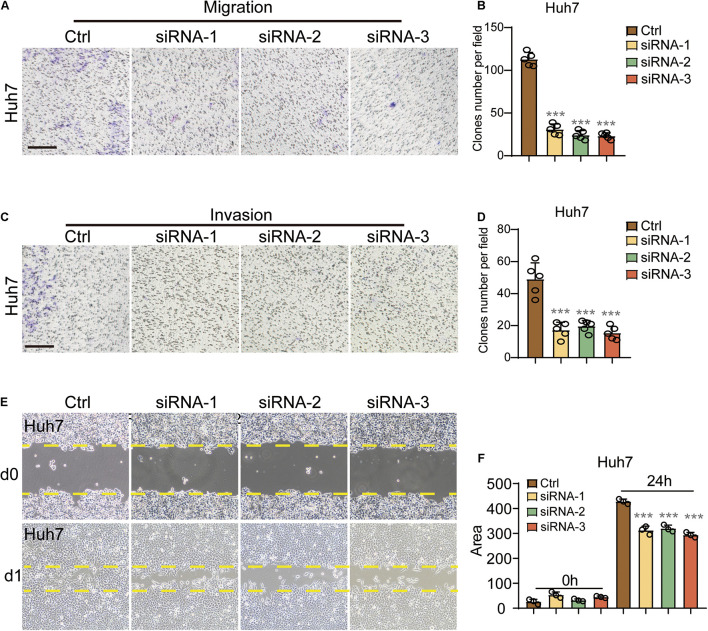
PGLS regulated the migration and invasion of HCC. **(A)** Representative images of CFUs in migration assay. **(B)** CFU clone number per field on Huh7 in migration assay. Invaded cells from five representative fields were counted. **(C,D)** The number of invasive cells significantly decreased after the treatment of these three siRNAs, respectively (*n* = 5). **(E)** Wound healing in Huh7 cells treated with siRNA-1,siRNA-2, and siRNA-3. The lines indicated the edge of wound at 0 and 24 h. **(F)** Migration rate was analyzed and expressed as the area of cells migrating from the original wounds. Data show individual values and mean ± SD. **(B,D,F)** Unpaired two-tailed Student *t*-tests, assessed statistical significance, ***p < 0.001.

In the migration assay, the two cell lines presented significantly fewer clones per field in the three KD groups as compared to the control group (Figures 4A,B and Supplementary Figures 2A,B). To analyze the effects of siRNA-1, siRNA-2, and siRNA-3 on HCC cell invasion, chamber invasion assay was performed on Hep3b and Huh7 cells. Significantly fewer cell clones were shown in the three siRNA groups, in both Hep3b and Huh7 cells (Figures 4C,D and Supplementary Figures 2C,D).

To further confirm that PGLS could regulate the migration of HCC. Wound-healing assay was performed, and the area covered by treated Huh7 cells was calculated by ImageJ. Nearly the same area was occupied at 0 h among four groups, and less migration length was shown in all three KD groups at 24 h as compared to the control group at the same time point (Figures 4E,F). Similar inhibitory effects were also observed in Hep3b cells (Supplementary Figures 2E,F).

### Pentose Phosphate Pathway Inhibitor 6-Aminonicotinamide Functionally Attenuated Hepatocellular Carcinoma Migration and Invasion

**FIGURE 5:**
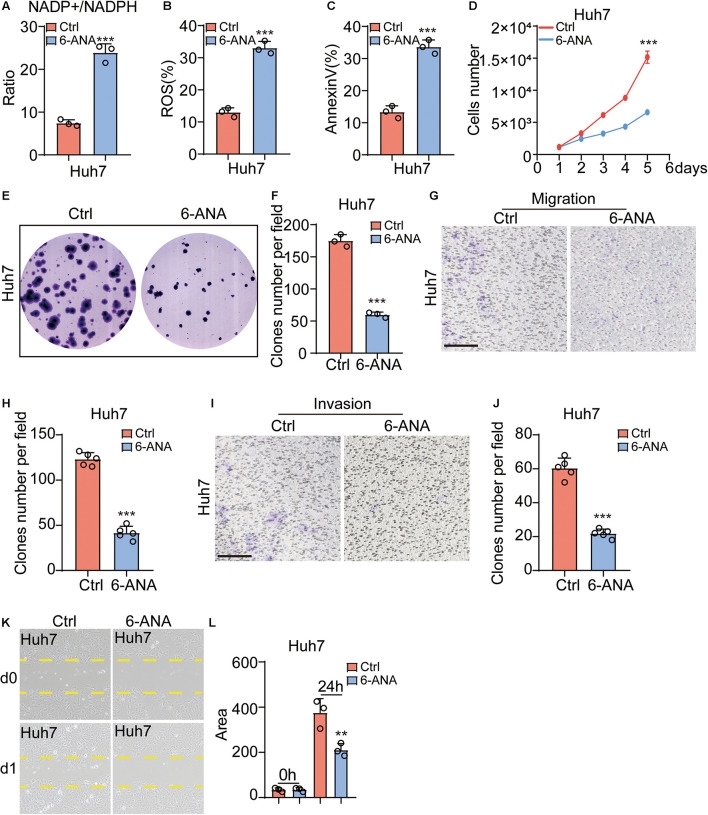
PPP pathway inhibitor 6-ANA functionally attenuated HCC migration and invasion. **(A)** The NADP^+^/NADPH ratio increased in the 6-ANA treated groups (*n* = 3). **(B,C)** The percentage of ROS and annexin V–positive cells increased as compared to the control group (*n* = 3). **(D)** The extent of cell proliferation was significantly reduced in the 6-ANA–treated group. **(E,F)** 6-ANA effectively suppressed the size and number of CFUs (*n* = 3). **(G,H)** Representative photographs showing the HCC cell lines that had passed through the well bottom to the lower surface of the membrane. The cells from five representative fields were counted. **(I,J)** Representative photographs showed the invasive cells that had passed through Matrigel to the lower surface of the membrane. Invaded cells from five representative fields were counted. **(K,L)** Migration rate was analyzed and expressed as the area of cells migrating from the original wounds. Data show individual values and mean ± SD. **(A–D,F,H,J,L)** Unpaired two-tailed Student *t*-tests, assessed statistical significance, **p < 0.01, ***p < 0.001.

6-ANA is a PPP inhibitor (Street et al., 1997; Arbe et al., 2020; Cheng et al., 2020). When Hep3b and Huh7 were cultured with a 10 μM 6-ANA concentration, we found that the NADP^+^/NADPH ratios increased significantly (Figure 5A and Supplementary Figure 3A), and the level of ROS and apoptosis rate were also increased in both HCC cell lines as compared to the control group (Figures 5B,C and Supplementary Figures 3B,C). Then we analyzed the effect of 6-ANA on Hep3b and Huh7 cell on proliferation. Similar to previous results, 6-ANA significantly suppressed cell proliferation in 10 μM concentration (Figure 5D and Supplementary Figure 3D). Subsequently the CFU results showed 6-ANA could also effectively reduce the size and number of CFUs in the treated group (Figures 5E,F and Supplementary Figures 3E,F). To test the effects of 6-ANA on HCC cell line migration, we did both chamber assay and wound-healing assay. The results showed that 6-ANA could reduce the clone number and migration area of Hep3b and Huh7 cells (Figures 5G,H,K,L and Supplementary Figures 3G,H,K,L). The number of invasive cells was also markedly decreased after the treatment of 6-ANA (Figures 5I,J and Supplementary Figures 3I,J). Overall, our data showed that PGLS was essential for the development of HCC.

## Discussion

In this study, by analyzing the liver single-cell sequencing data of HCC patients and healthy people, we were surprised to find an undifferentiated HCC population with high PGLS-specific expression. In the selected cells with high PGLS expression in human liver cancer tissues, we found the PPP pathway activated, and ROS production and HCC apoptosis reduced. PGLS inhibition inhibited the metabolic reprogramming of HCC cell lines. For the purpose of clinical transformation, we used 6-ANA to inhibit the PPP and inhibit the proliferation, clonal formation, migration, and invasion of HCC cell lines.

More and more evidences show that metabolism-related genes (HK2, FBP1, and PKM2) are very important for the occurrence and the development of HCC, and these genes promote the growth of HCCs by promoting the transcription of oncogenes (Chen et al., 2017; DeWaal et al., 2018; Li Q. et al., 2019; Hou et al., 2020). Our project analyzed the single-cell sequencing technology data of HCC patients and found that carbon metabolism played an important role in the differentiation of HCCs, and PGLS was the most obvious change among the genes that differ in carbon metabolism. Previous reports have shown that PGLS has a significant correlation with the occurrence of breast cancer, but its relationship with HCC has not been reported (Sivaraksa and Lowe, 2008), whereas Huh7 and Hep3b cell lines that knock down PGLS have decreased proliferation and metastasis and increased apoptosis in HCC cells. Thus, inhibiting PGLS in HCCs could be a novel strategy to inhibit HCC proliferation.

Metabolic reprogramming has been recognized as a hallmark of HCC (Kowalik et al., 2017; Jin and Zhou, 2019). Although metabolism-related drugs are currently approved as molecular targeting agents for HCC, their effect on life expectancy is generally limited (Rudalska et al., 2014). In this study, for the first time, we found that the specific high expression of PGLS in HCC activates the PPP pathway and reduces cell apoptosis induced by oxidative stress injury. In hepatocytes, low levels of PGLS lead to low activity of the PPP pathway. At this point, cells mainly rely on oxidative phosphorylation and glycolysis for energy. However, the rapid proliferation of HCCs requires activation of the PPP pathway to generate large amounts of R5P and NADPH, which are vital for the survival and proliferation of HCCs. R5P is the cornerstone for nucleic acid synthesis (Andriotis and Smith, 2019). NADPH is essential for anabolic reactions and redox equilibrium. This shift in metabolic patterns is critical for HCC growth.

## Conclusion

Finally, HCC patients with high PGLS expression have a poor prognosis. Interestingly, ROS levels and NADP^+^/NADPH levels were significantly reduced when we knocked down PGLS in Huh7 and Hep3b cell lines. Therefore, inhibition of PGLS can promote the recovery of the PPP metabolic profile of HCC, which may be a new way to regulate the metabolic reprogramming of HCC. Taken together, our data suggest that inhibition of PGLS may provide a novel strategy to achieve effective inhibition of HCC cells.

## Data Availability Statement

The datasets presented in this study can be found in online repositories. The names of the repository/repositories and accession number(s) can be found below: https://www.ncbi.nlm.nih.gov/geo/query/acc.cgi?acc=GSE149614.

## Ethics Statement

The studies involving human participants were reviewed and approved by the Third Affiliated Hospital of Sun Yat-sen University. Written informed consent to participate in this study was provided by the participants’ legal guardian/next of kin.

## Author Contributions

CL, JC, and YLi designed, performed most of the experiments, analyzed the data, and generated figures. YLi and BW contributed to single cell seq. ZY, XT, and YW contributed to bioinformatic analysis. ZH contributed to seahorse analysis. YP, HZ, and KY joined this project as rotation students for technique support. ZF and JX contributed for scientific discussion and manuscript preparation. YLu supervised the project and wrote the manuscript. All authors contributed to the article and approved the submitted version.

## Conflict of Interest

The authors declare that the research was conducted in the absence of any commercial or financial relationships that could be construed as a potential conflict of interest.

## Publisher’s Note

All claims expressed in this article are solely those of the authors and do not necessarily represent those of their affiliated organizations, or those of the publisher, the editors and the reviewers. Any product that may be evaluated in this article, or claim that may be made by its manufacturer, is not guaranteed or endorsed by the publisher.
