# Supplementary figures and images for: 6-Phosphogluconolactonase Promotes Hepatocellular Carcinogenesis by Activating Pentose Phosphate Pathway
Source: Front Cell Dev Biol. 2021 Oct 26;9:753196. doi: 10.3389/fcell.2021.753196 (PMC8576403; doi:10.3389/fcell.2021.753196)

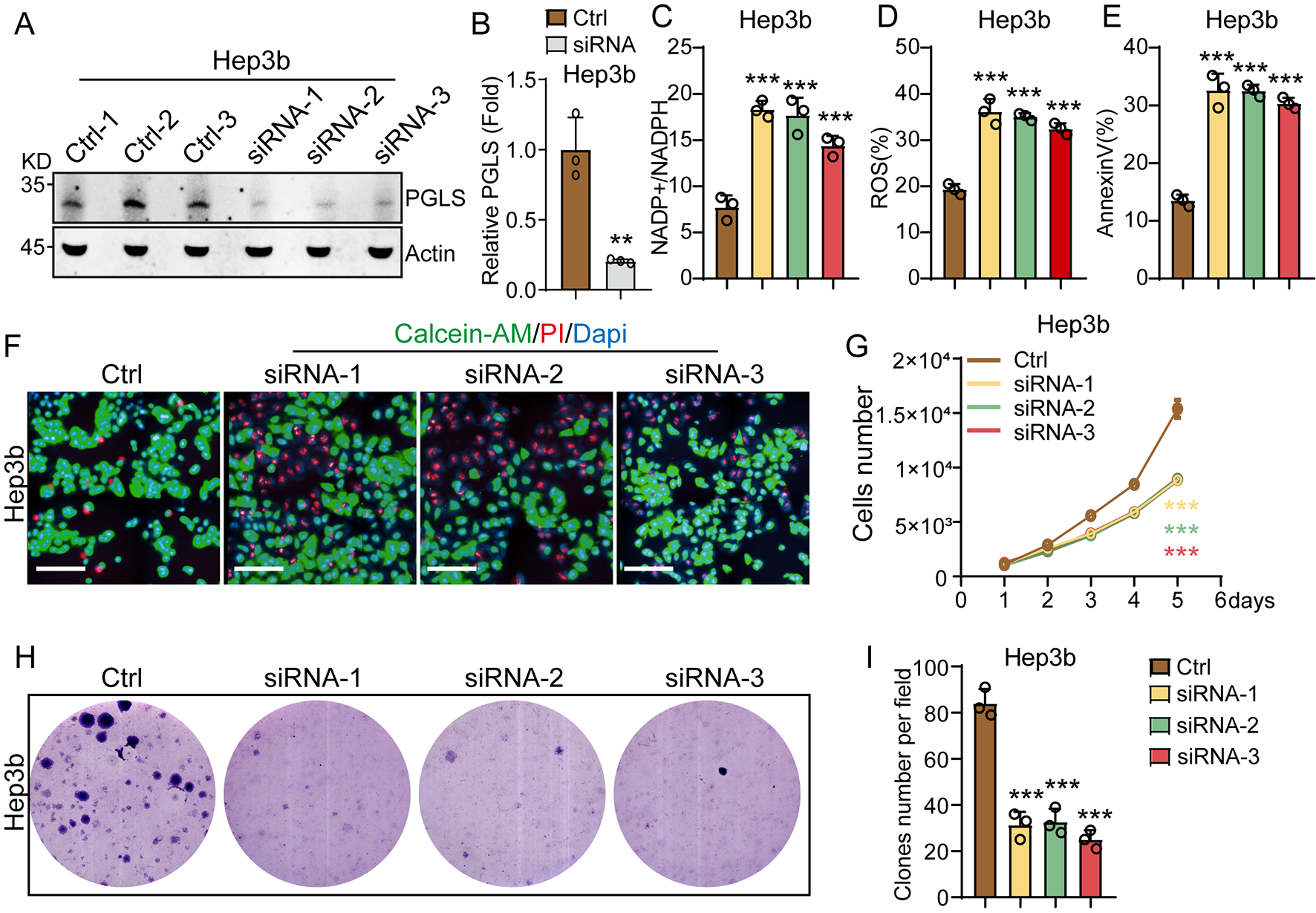

Supplement: Supplementary file 1 [file Image_1.TIF]

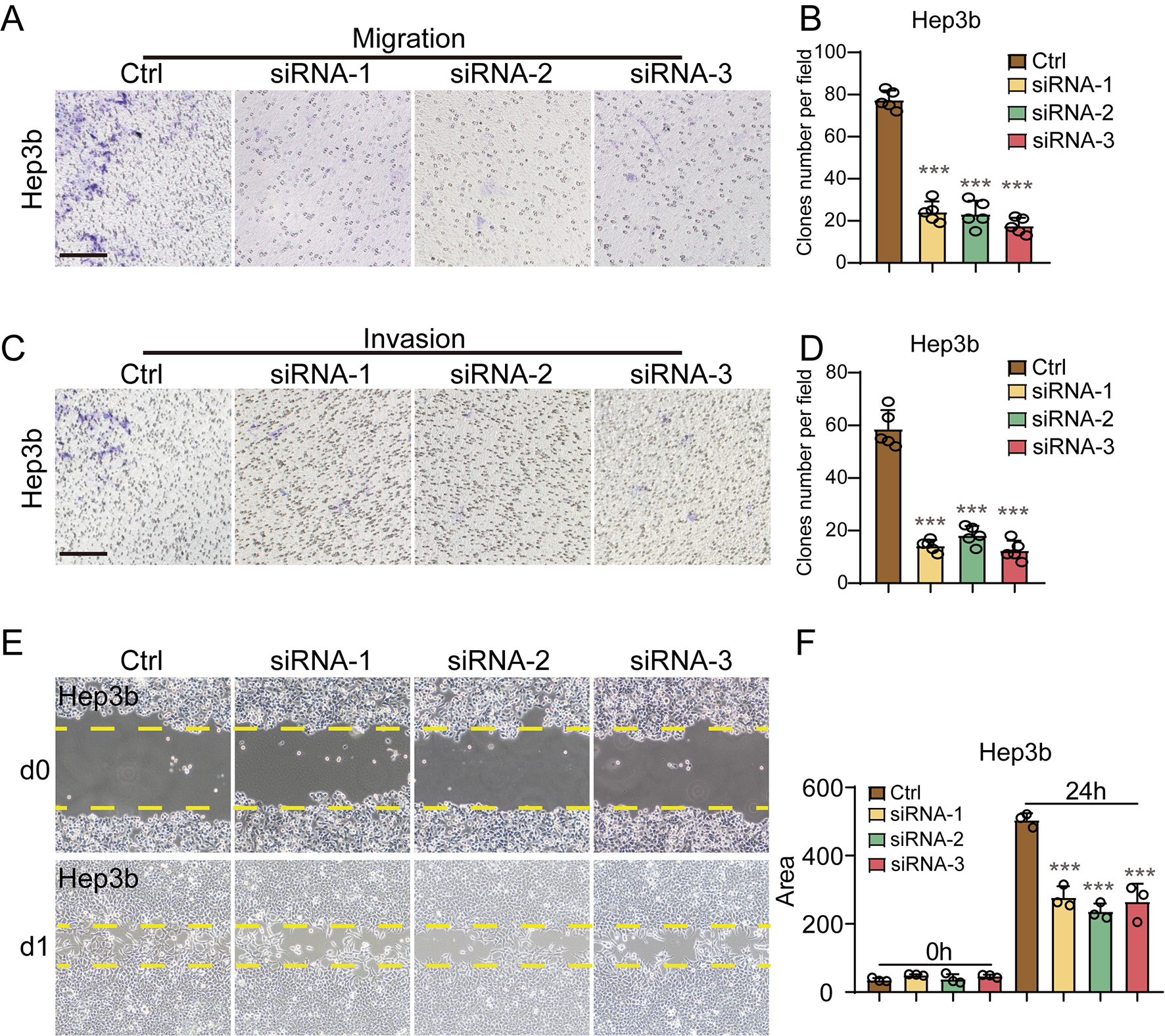

Supplement: Supplementary file 2 [file Image_2.TIF]

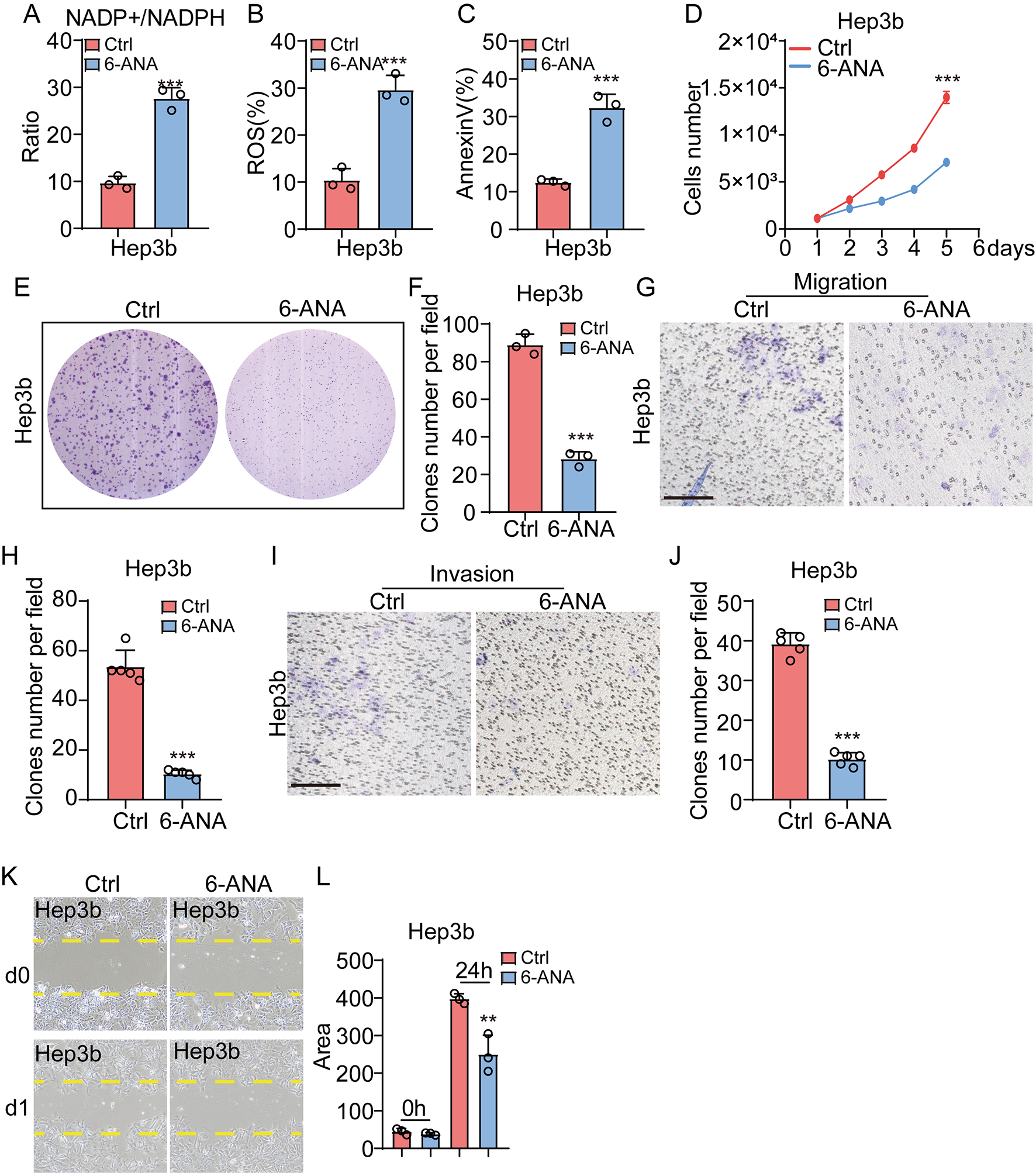

Supplement: Supplementary file 3 [file Image_3.TIF]
