# Supplementary material for: 6-Phosphogluconolactonase Promotes Hepatocellular Carcinogenesis by Activating Pentose Phosphate Pathway
Source: Front Cell Dev Biol. 2021 Oct 26;9:753196. doi: 10.3389/fcell.2021.753196 (PMC8576403; doi:10.3389/fcell.2021.753196)
Supplement: Supplementary file 4 [file Table_1.docx]

| Gene | Primer | Sequence (5’-3’) |
| --- | --- | --- |
| PGLS (Human) | Forward | GGAGCCTCGTCTCGATGCTA |
|  | Reverse | GAGAGAAGATGCGTCCGGT |
| GPC3 (Human) | Forward | CCTTTGAAATTGTTGTTCGCCA |
|  | Reverse | CCTGGGTTCATTAGCTGGGTA |
| CD24 (Human) | Forward | CTCCTACCCACGCAGATTTATTC |
|  | Reverse | AGAGTGAGACCACGAAGAGAC |
| MDK (Human) | Forward | CGCGGTCGCCAAAAAGAAAG |
|  | Reverse | TACTTGCAGTCGGCTCCAAAC |
| βActin (Human) | Forward | CATGTACGTTGCTATCCAGGC |
|  | Reverse | CTCCTTAATGTCACGCACGAT |

**Table 1. The PCR primer sequences used in this study**

**Table S1. The website of the raw data in this study**

The raw data of this study have been uploaded on the website:

https://www.jianguoyun.com/p/DUoWB44Q7L_cCRjWgIUE
