# Supplementary material for: 6-Phosphogluconolactonase Promotes Hepatocellular Carcinogenesis by Activating Pentose Phosphate Pathway
Source: Front Cell Dev Biol. 2021 Oct 26;9:753196. doi: 10.3389/fcell.2021.753196 (PMC8576403; doi:10.3389/fcell.2021.753196)
Supplement: Supplementary file 5 [file Data_Sheet_1.docx]

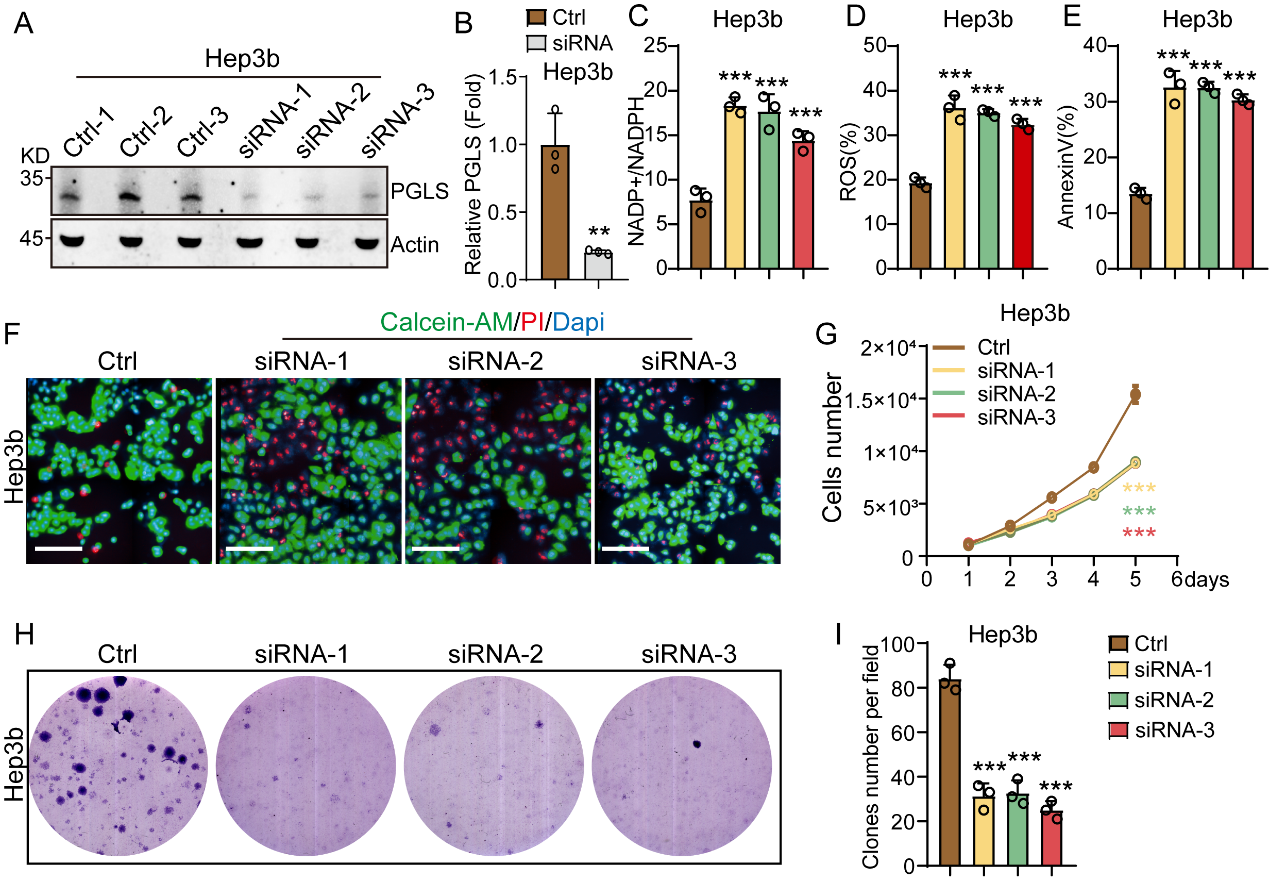


**Supplementary Figure 1**. **PGLS regulated the proliferation and apoptosis of HCC.**

(A) Transfection with the three siRNAs could effectively knock down the expression of PGLS on Hep3b. (B) Quantitative analysis of western blot in (A). (C) The ratio of NADP+/NADPH on Hep3b. (D) The percentage of ROS on Hep3b. (E) The apoptosis rate on Hep3b. (F) Fluorescence micrographs of Hep3b after staining with Calcein-AM, PI and Dapi. (G) Proliferation curve of Hep3b transfected by siRNA-1, siRNA-2 and siRNA-3. (H) Representative field of CFU, forming by Hep3b treated with siRNA-1, siRNA-2 and siRNA-3. (I) CFU clone number per field on Hep3b. Data show individual values and mean ± s.d. B-E, G and I, unpaired two-tailed Student’s t-tests, assessed statistical significance, * P<0.05, ** P<0.01, *** P<0.001.


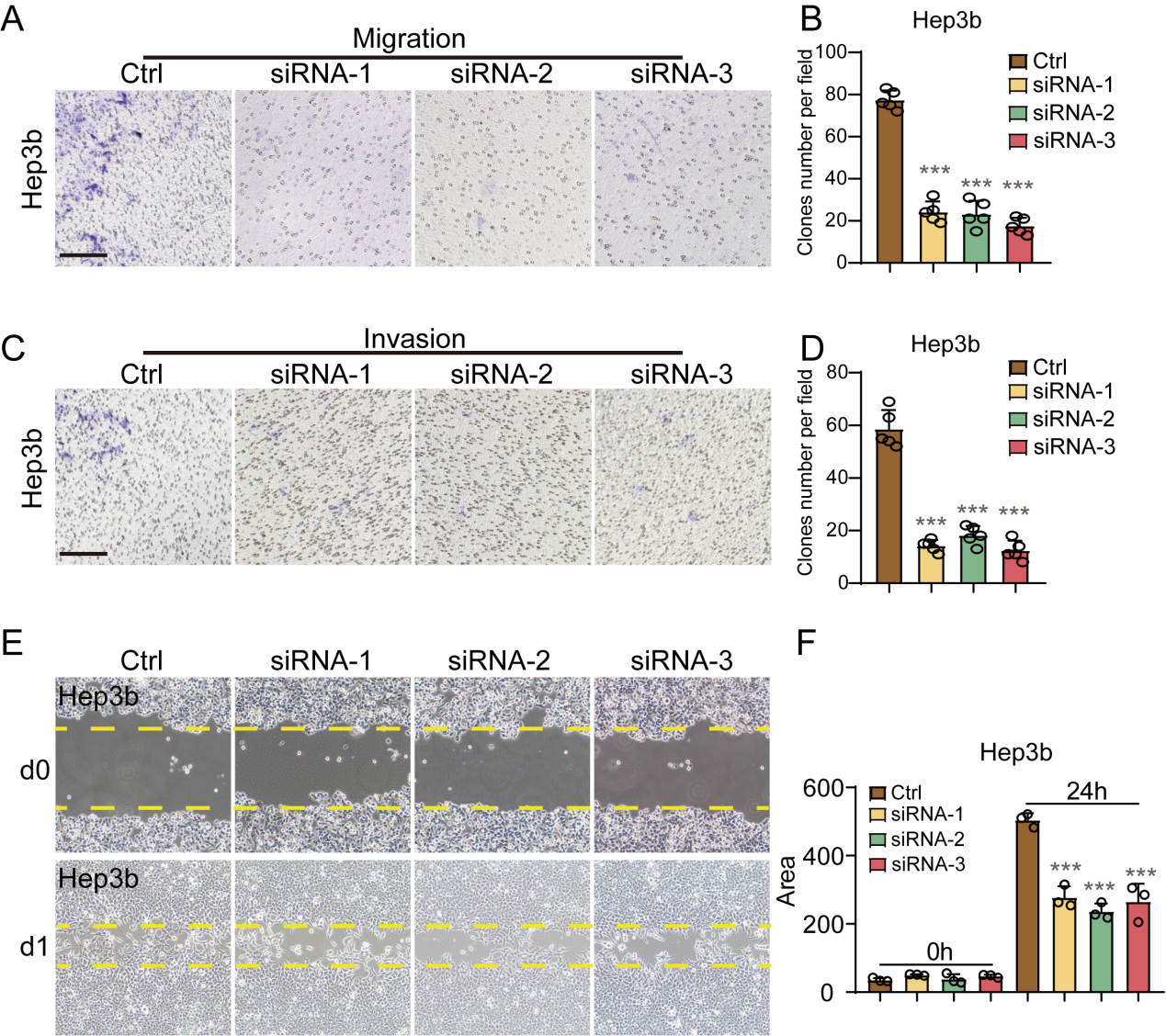


**Supplementary Figure 2**. **PGLS regulated the migration and invasion of HCC.**

(A) Representative image of CFU in migration assay on Hep3b. (B) CFU clone number per field on Hep3b in migration assay. Invaded cells from 5 representative fields were counted. (C-D) The number of invasive cells significantly decreased after the treatment of these three siRNAs respectively (n = 5). (E) Wound healing in Hep3b cells treated with siRNA-1, siRNA-2, siRNA-3. The lines indicated the edge of wound at 0 h and 24h. (F) Migration rate was analyzed and expressed as the area of cells migrating from the original wounds. Data show individual values and mean ± s.d. B, D and F, unpaired two-tailed Student’s t-tests, assessed statistical significance, * P<0.05, ** P<0.01, *** P<0.001.


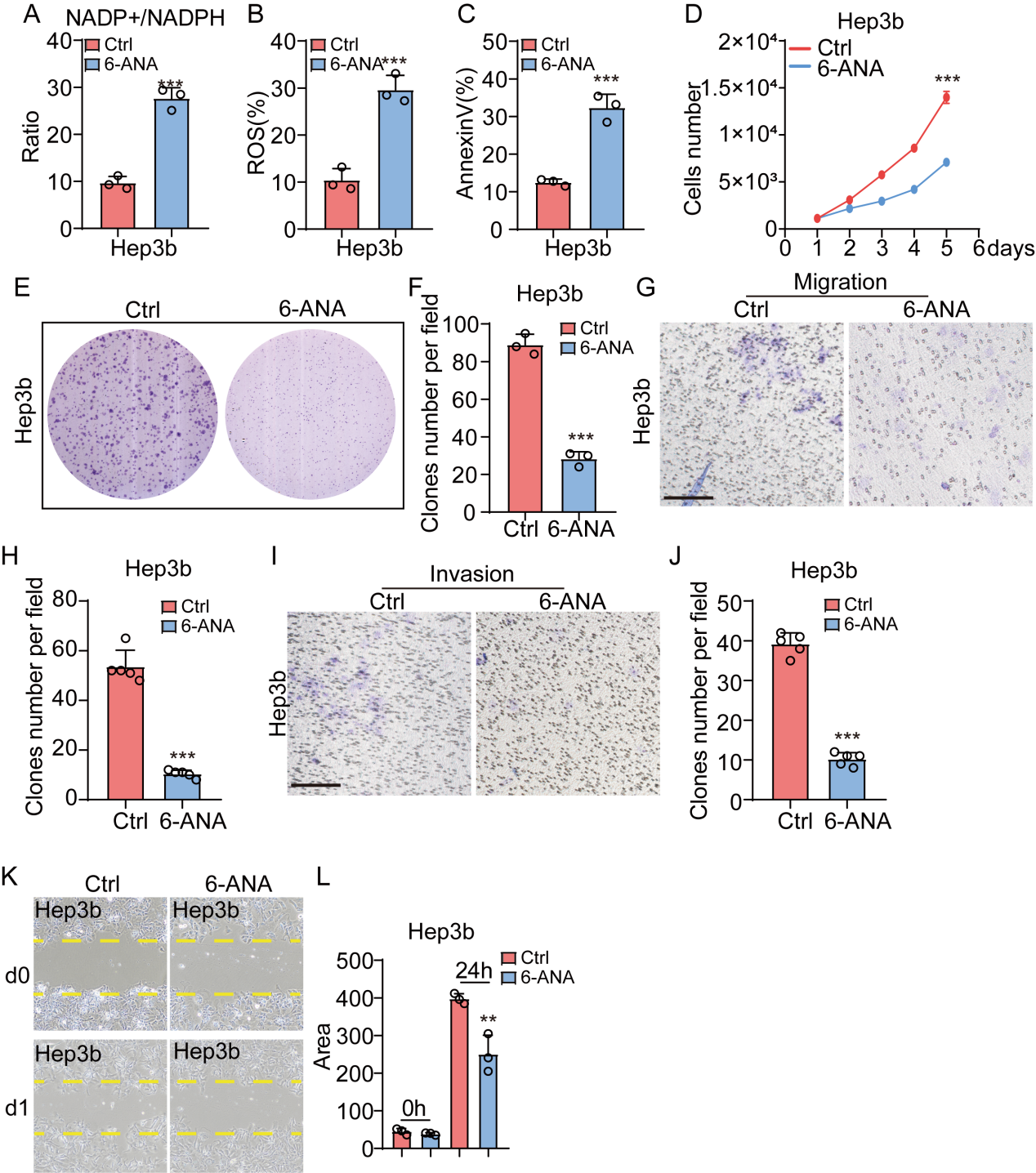


**Supplementary Figure 3**. **PPP pathway inhibitor 6-ANA functionally attenuated HCC migration and invasion in Hep3b.** (A) The ratio of NADP+/NADPH increased in the 6-ANA treated groups (n = 3). (B-C) The percentage of ROS and Annexin V positive cells increased as compared to the control group (n = 3). (D) The extent of cell proliferation was significantly reduced in the 6-ANA treated group. (E-F) 6-ANA effectively suppressed the size and number of CFU (n = 3). (G-H) Representative photographs showing the HCC cell lines that had passed through the well bottom to the lower surface of the membrane. The cells from 5 representative fields were counted. (I-J) Representative photographs showing the invasive cells that had passed through matrigel to the lower surface of the membrane. Invaded cells from 5 representative fields were counted. (K-L) Migration rate was analyzed and expressed as the area of cells migrating from the original wounds (n = 3), Data show individual values and mean ± s.d. A-D, F, H, J and L, unpaired two-tailed Student’s t-tests, assessed statistical significance, * P<0.05, ** P<0.01, *** P<0.001.
